# Monocytic myeloid-derived suppressor cells from females, but not males, alleviate CVB3-induced myocarditis by increasing regulatory and CD4^+^IL-10^+^ T cells

**DOI:** 10.1038/srep22658

**Published:** 2016-03-04

**Authors:** Nan Su, Yan Yue, Sidong Xiong

**Affiliations:** 1 Jiangsu Key Laboratory of Infection and Immunity, Institutes of Biology and Medical Sciences, Soochow University, Suzhou, P.R. China

## Abstract

Coxsackievirus group B type 3 (CVB3) is a common etiologic agent of viral myocarditis and often causes sexually dimorphic myocarditis with increased incidence and mortality in male. So far, the underlying mechanism for the high male prevalence is not well elucidated. In this study, we deciphered the role of myeloid-derived suppressor cells (MDSCs) in the gender bias in murine CVB3-induced myocarditis by comparing their frequencies, subsets as well as immune suppressive functions. We found that much more myocardial MDSCs were enriched in infected females than males, with dramatically higher percentage ratio of CD11b^+^Ly6G-Ly6C^high^ monocytic subset (M-MDSCs) to CD11b^+^Ly6G^+^Ly6C^low^ granulocytic subset (G-MDSCs). Interestingly, more potent suppression on T cell proliferation was also evidenced in female-derived M-MDSCs. Consistently, adoptive transfer of female- but not male-derived M-MDSCs efficiently alleviated CVB3-induced myocarditis in male recipient mice, and this protection could be ascribed to the increased induction of regulatory and CD4^+^IL-10^+^ T cells. Our study suggested that myocardial MDSCs were distinctively induced not only in quantities but also in phenotypes and immune suppressive functions in CVB3-infected males and females; and female-derived more suppressive M-MDSCs contributed to their insensitivity to CVB3-induced myocarditis.

Viral myocarditis is a major cause of sudden death in infants and young adults under 40 years, and can further develop into dilated cardiomyopathy (DCM) and congestive cardiac failure^1^,^2^,^3^. Enteroviruses, especially coxsackieviruses, have been considered as the most common cause of viral myocarditis^4^,^5^,^6^. It is well established that sex and sex-associated hormones contribute to the susceptibility of CVB3-induced myocarditis^7^,^8^,^9^. Although similar viral infection efficiency has been measured in both genders, the estimated incidence of myocarditis in men is 2-fold or more than in women^10^. In murine models of CVB3-induced myocarditis, significantly higher occurrence and more severe myocardial inflammation are also noted in males^11^.

Increasing evidence has shown that indirect immune-mediated injury, but not direct virus-induced damage, is the prominent pathological mechanism of CVB3-induced myocarditis^12^,^13^. Two genders display diverse immune patterns, strength as well as immune modulations in the disease process of viral myocarditis^14^,^15^. It has been showed that in CVB3-infected male mice, significantly increased Toll-like receptor 4 (TLR4) led to the augmented IL-1β/IL-18 expression and reduced regulatory T (Treg) cells production; while in female, up-regulated T cell immunoglobulin mucin-3 (Tim-3) resulted in the increased expression of regulatory molecule CTLA-4 on CD4^+^ T cells and higher frequency of Treg cells^11^. Our previous study showed that myocardial macrophage polarization also contributed to the gender bias of viral myocarditis in mice. Type I phenotype of macrophage dominated in infected males and aggravated myocardial inflammation. While type II phenotype of macrophage dominated in infected females and preferentially alleviated myocarditis by inducing Treg^16^. These literatures suggest that various immune modulation mechanisms contribute a lot to the gender bias of CVB3-induced myocarditis.

Myeloid-derived suppressor cells (MDSCs) have been described as a heterogeneous cell population consisting of myeloid progenitor cells and immature cells with potent immune suppressive function^17^,^18^. Besides of their particular roles in tumor immune escape^3^,^19^, the functions of MDSCs in infection and inflammation have also attracted more and more attention^20^,^21^,^22^,^23^. It has been proven that viruses could efficiently recruit a large amount of tissue MDSCs to inhibit host defense and benefit their infection^24^,^25^,^26^. In verse, induced MDSCs could modulate the pattern and strength of innate and adaptive immune responses to avoid massive tissue immunopathology and inflammation^27^,^28^. Interestingly, a previous study revealed that in *Trypanosoma cruzi-*induced myocarditis, abundant MDSCs accumulated in heart tissues and facilitated infection by suppressing T cell proliferation^29^, indicating the participation of MDSCs in infectious heart diseases. Therefore, MDSCs may exert versatile functions in infectious disease courses, and elucidating the role of MDSCs in the CVB3-induced myocarditis may help us gain a better understanding of the mechanisms underlying the gender bias of viral myocarditis.

In this study, the frequency and phenotype of MDSCs were characterized in male and female mice with CVB3-induced myocarditis, and their potential roles in the development of viral myocarditis were deciphered.

## Results

### Females revealed less susceptibility to CVB3-induced myocarditis than males

**Figure 1:**
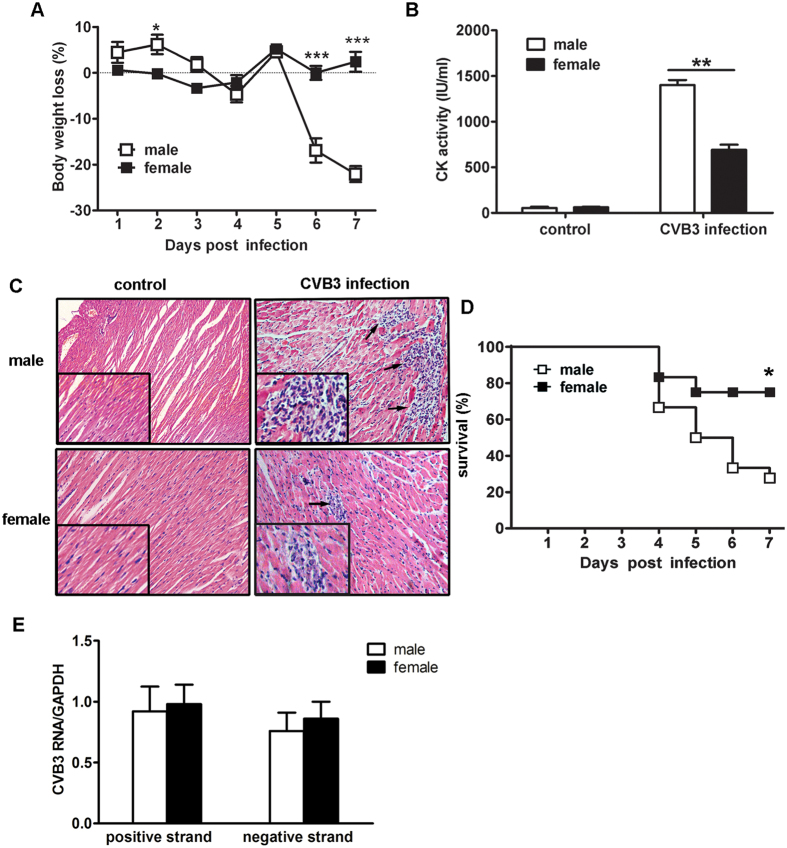
Sex differences in susceptibility to CVB3-induced myocarditis. (**A**) Body weight changes of infected mice in a 7-day period. (**B**) Serum CK activity at day 7. (**C**) Myocardial histopathological alteration at day 7 (Arrows indicated infiltrating inflammatory cells). (**D**) Survival rate in a 7-day period. (**E**) Myocardial viral loads at day 7. Each group contained 5 mice. For survival rate evaluation, each group contained 16 mice. Individual experiment was conducted 3 times with similar results, and the representative data were shown. *p < 0.05, **p < 0.01, ***p < 0.001.

Age-matched male and female BALB/c mice were intraperitoneally infected with CVB3. Seven days later, the incidence and severity of myocarditis were measured and compared in both genders. As illustrated in Fig. 1, females exhibited slighter body weight loss, lower serum CK activity as well as limited myocardial inflammatory infiltration (indicated by the arrows, Fig. 1C) compared with males, indicating less severe viral myocarditis. In consistence with limited myocardial inflammation and injury, the 7-day survival rate of females achieved 80%, significantly higher than 30% of males (*p* = 0.01, Fig. 1D). These results confirmed our previous observation that female mice showed less susceptibility to CVB3-induced myocarditis^16^. In addition, this gender bias was not attributed to the less efficient CVB3 replication in females, since similar myocardial viral loads were detected on day 7 post infection in both genders (Fig. 1E).

### More myocardial MDSCs were enriched in females than males with CVB3-induced viral myocarditis

**Figure 2:**
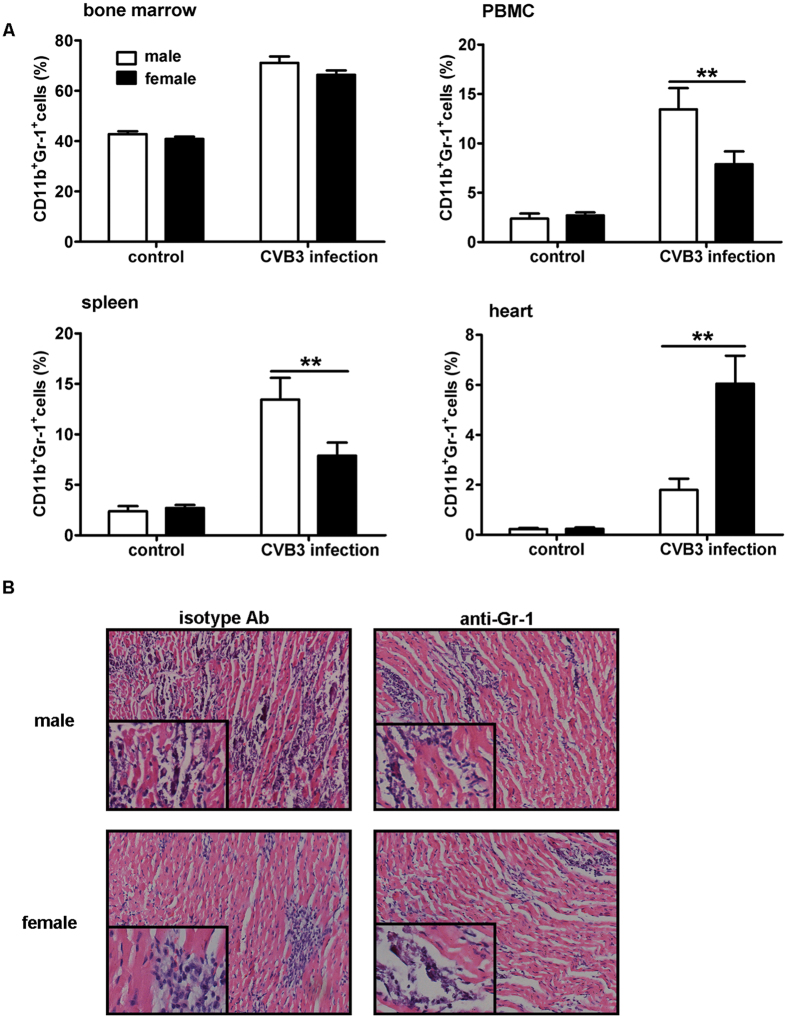
Frequency of MDSCs in CVB3-infected male and female mice. (**A**) Seven days post CVB3 infection, frequencies of CD11b^+^Gr-1^+^ MDSCs in bone marrow, PBMC, spleen and heart tissues were measured in male and female mice by flow cytometry. Data were presented as the means ± SEM of 5 mice per group. (**B**) Myocardial histopathological alteration in infected mice receiving anti-Gr-1 antibody treatment. Individual experiment was conducted 3 times with similar results, and the representative data were shown. **p < 0.01.

Mounting evidence suggests that different patterns and potencies of immune responses between males and females cause the dimorphic sensitivity to viral myocarditis^15^,^30^. Therefore, we speculated that the frequency and/or function of immune regulatory cells may be distinct in both genders. MDSCs represent myeloid originated immunoregulatory cells with immune suppressive functions on both innate and adaptive immunity stages^17^,^31^. To evaluate the influence of MDSCs on sex-based sensitivity to viral myocarditis, their frequencies in CVB3-infected male and female mice were determined. As shown in Fig. 2, no distinct sex difference in MDSCs percentages was seen in naive mice. Following CVB3 infection, the frequencies of MDSCs in the spleen and blood significantly increased in males and were much higher than females. However, the situation was completely opposite in the heart tissues, the frequency of MDSCs reached 5.4% in females, robustly higher than 1.8% in males (Fig. 2A). Besides, depletion of MDSCs with anti-Gr-1 antibody could obviously alleviate CVB3-induced myocarditis in males, but have marginal impact in females (Fig. 2B).

### Monocytic MDSCs (M-MDSCs) predominantly distributed in the myocardial tissue of infected females

**Figure 3:**
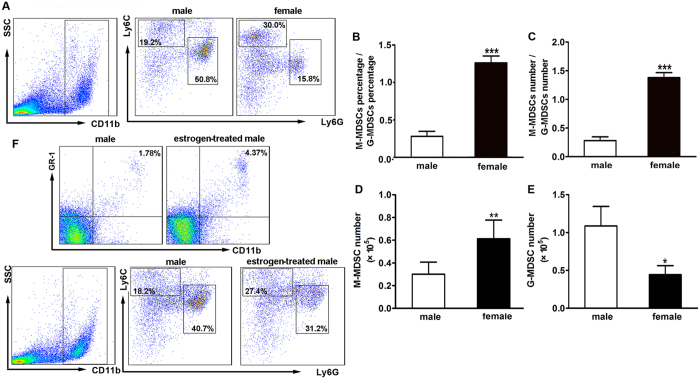
More M-MDSCs distributed in the hearts of infected female mice. (**A**) Percentages of CD11b^+^Ly6G^–^Ly6C^high^ M-MDSCs and CD11b^+^Ly6G^+^Ly6C^low^ G-MDSCs in the hearts of CVB3-infected mice. (**B**) Percentage ratio of myocardial M-MDSCs to G-MDSCs. (**C**) Number Ratio of myocardial M-MDSCs to G-MDSCs. (**D**) Absolute number of myocardial M-MDSCs. (**E**) Absolute number of myocardial G-MDSCs. Data were presented as the means ± SEM of 5 mice per group. (**F**) Percentages of CD11^+^Gr-1^+^ MDSCs, G-MDSCs and M-MDSCs in the estrogen-treated males post CVB3 infection. Individual experiment was conducted 3 times with similar results, and the representative data were shown.*p < 0.05, **p < 0.01, ***p < 0.001.

MDSCs consist of two major populations: CD11b^+^Ly6G^+^Ly6C^low^ granulocytic (G-MDSCs) and CD11b^+^Ly6G^-^Ly6C^high^ monocytic (M-MDSCs) cells, we next detected these two subsets in the heart tissues of both genders. As shown in Fig. 3A and B, 18.2% M-MDSCs and 55.2% G-MDSCs were observed in males, with M-MDSCs/ G-MDSCs ratio to be 0.3. While in females, M-MDSCs frequency dramatically increased to 31.2% and its ratio to G-MDSCs augmented to 1.3, indicating M-MDSCs as the predominantly expanded subset in the hearts of infected females. Consistently, the absolute number of myocardial M-MDSCs in females was considerably larger than that in males (Fig. 3D). The situation of myocardial G-MDSCs was totally opposite, the number of G-MDSCs was obviously higher in males (Fig. 3E). To further investigate whether estrogen play a role in MDSCs infiltration and phenotypes, CVB3-infected male mice were treated with 17β-estradial and then total and two subsets of myocardial MDSCs were detected. As shown in Fig. 3F, estrogen could obviously promote the enrichment of CD11b^+^Gr-1^+^ MDSCs post CVB3 infection, with the priority to increase the percentage of CD11b^+^Ly6G^–^Ly6C^high^ monocytic (M-MDSCs). Our results were further supported by the previous study which reported that 17β-estradiol promote the accumulation of myeloid-derived suppressor cells^30^.

**Figure 4:**
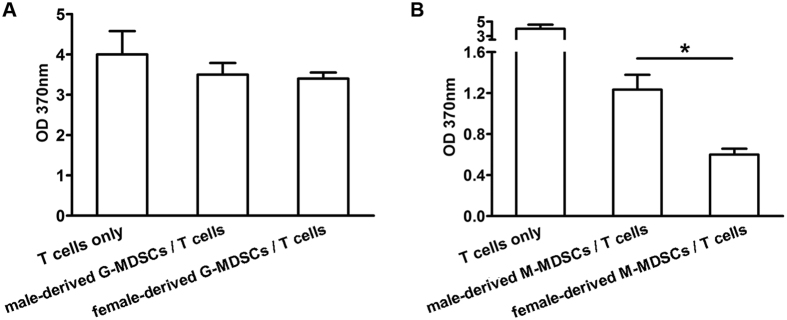
Female-derived myocardial M-MDSCs possessed more potent suppressive function on T cell proliferation. (**A**) Suppressive function of myocardial G-MDSCs on T cell proliferation. (**B**) Suppressive functions of myocardial M-MDSCs. Data were collected from the pool of 8–10 mice for each group. Individual experiment was conducted 3 times with similar results, and the representative data were shown. *p < 0.05.

Next, immune suppressive functions of myocardial MDSCs subsets were compared in both genders. As shown in Fig. 4, T cell proliferation provoked by anti-CD3 and anti-CD28 stimulation was not obviously influenced by both male- and female-derived G-MDSCs, but dramatically inhibited by female-, but not male-derived M-MDSCs. These data indicated that myocardial M-MDSCs subset possessed the immune suppressive function in the context of CVB3-induced myocarditis; meanwhile, female-derived myocardial M-MDSCs dominated not only in quantity but also in immune suppressive function compared with their counterpart in males.

### Adoptive transfer of female-derived M-MDSCs significantly alleviated CVB3-induced myocarditis in males

**Figure 5:**
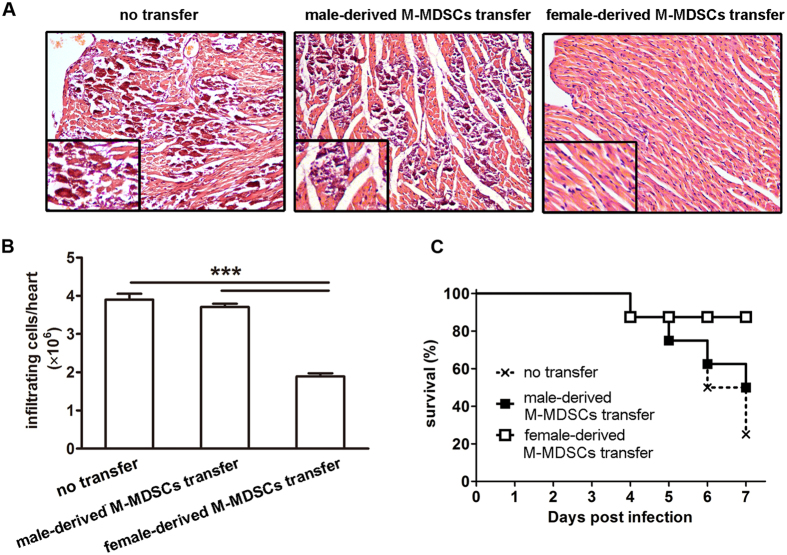
Adoptive transfer of female-derived M-MDSCs significantly alleviated CVB3-induced myocarditis in male recipients. CVB3-infected male mice received female-derived M-MDSCs on day 3, 4 days later, myocardial histopathological alteration (**A**), infiltrating inflammatory cell number (**B**) as well as a 7-day-period survival rate (**C**) were examined. Data were presented as the means ± SEM of 5 mice per group. For survival rate evaluation, each group contained 8 mice. Individual experiment was conducted 3 times with similar results, and the representative data were shown. ***p < 0.001.

**Figure 6:**
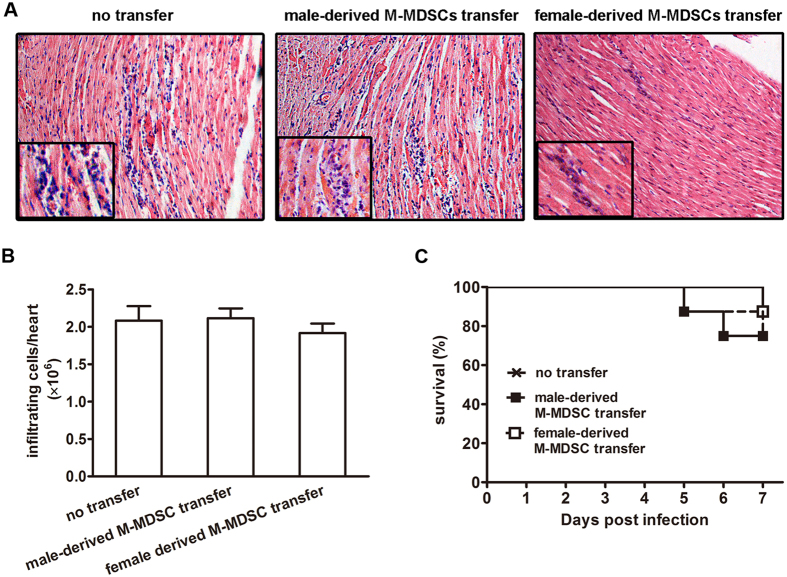
Adoptive transfer of male-derived M-MDSCs had no significant influence on CVB3-induced myocarditis in female recipients. CVB3-infected female mice received male-derived M-MDSCs on day 3, 4 days later, myocardial histopathological alteration (**A**), infiltrating inflammatory cell number (**B**) as well as a 7-day-period survival rate (**C**) were examined. Data were presented as the means ± SEM of 5 mice per group. For survival rate evaluation, each group contained 8 mice.

To further assess the immune suppressive ability of female-derived M-MDSCs *in vivo* and evaluate their role in viral myocarditis, M-MDSCs from CVB3-infected female mice were enriched by FACS sorting and adoptively transferred into infected recipient male mice on day 3, and myocarditis severity was evaluated 4 days later. In contrast to untransferred mice, males transferred with female-derived M-MDSCs exhibited much restricted myocardial inflammation foci (Fig. 5A) as well as fewer inflammatory cells (Fig. 5B), indicating the efficient alleviation of viral myocarditis. Consistently, the cumulative 7-day survival rate also enhanced from 25% to 87.5%, although no statistically significant difference was evidenced compared with males receiving male-derived M-MDSCs or no transfer (Fig. 5C). These data indicated that only female- but not male-derived M-MDSCs efficiently ameliorated CVB3-induced myocardial inflammation. In sharp contrast, no significantly changed myocarditis was shown in female recipients following male-derived M-MDSCs (Fig. 6).

### Female-derived M-MDSCs transfer potently increased regulatory and CD4^+^IL-10^+^ T cells in CVB3-infected males

**Figure 7:**
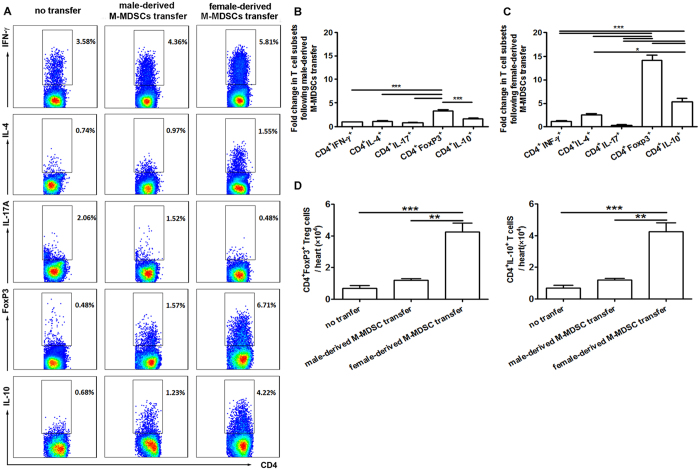
Adoptive transfer of female-derived M-MDSCs promoted regulatory and CD4^+^IL-10^+^ T cell production. CVB3-infected male mice received male- or female-derived M-MDSCs on day 3. (**A**) At day 7, the percentage of CD4^+^ T cells secreting IFN-γ, IL-4, IL-17 or IL-10 and the percentage of Treg cells in recipient mice were analyzed by flow cytometry. (**B**) Fold change in the percentage of T cell subsets in male recipient compared with those in mice receiving no transfer. (**C**) Absolute number of Treg and IL-10^+^CD4^+^ T cells in the hearts. Data were collected from the pool of 8–10 mice for each group. Individual experiment was conducted 3 times with similar results, and the representative data were shown. *p < 0.05, **p < 0.01, ***p < 0.001.

To elucidate the mechanism underlying the protective effect of female-derived M-MDSCs on CVB3-induced myocarditis, T cell responses in the recipient male mice were examined by FACS assays. As shown in Fig. 7A, adoptive transfer substantially raised CD4^+^Foxp3^+^ Treg frequency from 0.48–6.71%, about 14-fold higher than that of control mice; meanwhile, a 6-fold higher frequency was also achieved in CD4^+^IL10^+^ T cells, suggesting that female-derived M-MDSCs exerted their protection against viral myocarditis mainly via promoting regulatory and IL-10-secreting T cell production. In accordance with the frequency data, absolute numbers of myocardial Treg and CD4^+^IL10^+^ T cells robustly increased following female-derived M-MDSCs transfer, and were about 12-fold and 4-fold higher than the counterparts of control mice, respectively (Fig. 7C,D). In contrast, adoptive transfer of male-derived M-MDSCs had much less impact on CD4^+^ T cell subsets, although slight increase in Treg percentage and number were observed compared with control mice receiving no transfer, but far less than those in males receiving female-derived M-MDSCs (Fig. 7B,C). These data are also in agree with the limited therapeutic effect of male-derived M-MDSCs transfer (Fig. 6).

## Discussion

In the present study, we found that much more myocardial MDSCs were induced in female than male mice in CVB3-induced myocarditis, and a suppressive monocytic subset (M-MDSCs) was dominant in females, while a non-suppressive granulocytic subset (G-MDSCs) was dominant in males. In addition, female-derived cells possessed a more potent T cell suppressive function, and could efficiently relieve CVB3-induced myocarditis. Yet their male-derived counterpart had limited T cell suppressive capability and exerted no such protective effect.

MDSCs are a heterogeneous population of myeloid cells, broadly defined as CD11b^+^Gr-1^+^ cells that expand under the circumstances of tumor, infection, inflammation and autoimmune diseases. Several studies have proposed a link between the increased MDSCs and the development and prognosis of viral infectious diseases^32^,^33^,^34^. In line with that, here we observed a substantial enrichment of MDSCs in CVB3-infected mice, suggesting their possible involvement in the viral myocarditis. When compared with males, female mice had fewer splenic MDSCs, but much more myocardial MDSCs, suggesting distinct enrichment patterns of MDSCs in both genders. Depletion of MDSCs with anti-Gr-1 antibody (RB6-8C5, reacting with a common epitope on Ly-6G and Ly-6C) could significantly relieve myocarditis in males, which might be attributed to the depletion of cardiac dominant non-suppressive G-MDSCs subset (about 3-fold higher than M-MDSCs). However, anti-Gr-1 antibody treatment had marginal impact on myocarditis in females, which might be caused by the both deletion of immune-suppressive M-MDSCs (about 1.3-fold higher than G-MDSCs) and infiltrating G-MDSCs. According to the different epitopes in Gr-1, MDSCs have been subdivided into two subsets: monocytic (CD11b^+^Ly6G^–^ Ly6C^high^) and granulocytic (CD11b^+^Ly6G^+^Ly6C^low^) cells^35^,^36^,^37^. Until now, it is still controversy whether these two populations own similar biological functions or not. Some reports indicated that both M-MDSCs and G-MDSCs possess comparable immune suppressive potentials^38^,^39^, whereas others demonstrated that they may exert divergent activities in a series of diseases such as infectious and autoimmune diseases^40^,^41^, implying complicated roles of MDSCs in pathological processes. Herein, we found that in CVB3-infected mice, myocardial M-MDSCs were the predominant T cell proliferation suppressive subset, whereas G-MDSCs were not.

Further, we found that female-derived M-MDSCs possessed a more potent suppressive function on T cell proliferation than those from males. Following adoptive transfer, only female-derived M-MDSCs efficiently alleviated CVB3-induced myocarditis, reflected by limited loci of myocardial inflammation and enhanced survival rate, while no obviously improved myocarditis was seen in mice receiving male-derived M-MDSCs. Given the immunosuppressive function of male-derived M-MDSCs observed in *in vitro* assays, we attributed this inconsistent *in vivo* data to the current transfer schedule we applied. It turned out that only one time transfer of male-derived M-MDSCs at the dose of 2 × 10^6^ cells on day 3 post infection could not effectively alleviate CVB3-induced viral myocarditis; however it is reasonable to deduce that when increasing the transferring cell amounts and/or frequencies, or change the transferring time points, male-derived M-MDSCs should also exhibit obvious therapeutic effects on CVB3-induced myocarditis. Besides, we observed that following female-derived M-MDSCs transfer, myocardial viral load in male recipients increased slightly with no statistical significance. When transferring male-derived M-MDSCs to female, no noticeable improvement on viral myocarditis could be seen, reflected by the comparable pathological myocardial changes and survival rates, indicating that male-derived M-MDSCs had less immune suppressive and myocarditis-alleviating abilities. As many cytokines and molecules have been reported to influence the induction and suppressive function of tissue MDSCs and their subsets^40^,^42^,^43^, these different immune modulation functions of myocardial MDSCs in both genders may be attributed to their various myocardial environments. Moreover, female-derived M-MDSCs transfer could significantly increase Treg and CD4^+^IL-10^+^ T cell frequencies and absolute numbers, which might work together to better control myocardial immunopathological injury in CVB3-infected male mice^44^,^45^. Of course, herein we could not exclude the potential effects of MDSCs on innate immune responses following CVB3 infection, such as differential macrophage polarization which has been proven to play an important role in the sex difference in the sensitivity of CVB3-induced viral myocarditis^15^,^46^. In fact, a line of evidence suggest that under the tumor condition, MDSCs not only possess many features of M2 macrophages (such as expressing arginase 1 and NOS2), but also might function as the progenitors of tumor-associated macrophages^23^,^47^. In addition, MDSCs could further modulate macrophage mediated cytotoxicity^48^. Whether G-MDSCs or M-MDSCs could further differentiate into polarized macrophages or modulate macrophage functions in the context of CVB3-induced myocarditis need to be further interrogated. In summary, our findings suggest that compared with male mice, more abundant MDSCs dominated by monocytic phenotype and with more potent suppressive function were observed in the hearts of CVB3-infeced females, which provided protection against CVB3-induced myocarditis by increasing Treg and CD4^+^IL-10^+^ T cells. This study may not only help us better understand the role of MDSCs in CVB3-induced myocarditis, but also provide a new explanation for the gender bias in the viral myocarditis.

## Methods

### Mice and virus

Male and female BALB/c (H-2^d^) mice, 6-week-old, were purchased from Experimental Animal Centre of Chinese Academy of Sciences (Shanghai, P. R. China) and bred in the specific pathogen-free facility. All animal experiments were carried out in strict accordance with the recommendations in the Guide for the Care and Use of Medical Laboratory Animals (Ministry of Health, P. R. China, 1998). The protocol was approved by the Ethical Committee of Soochow University.

CVB3 (Nancy strain) was maintained by passage through Hela cells (ATCC number: CCL-2). Mice were infected by an intraperitoneal injection of 0.1 ml of PBS containing 1000 PFU doses of CVB3.

### Flow cytometry

Single-cell suspension of bone marrow (BM), splenocytes, peripheral blood mononuclear cells (PBMC) or myocardial inflammatory cells from CVB3-infected mice was prepared, and stained with the following monoclonal antibodies diluted with 1% FBS in PBS: FITC anti-mCD11b, PE anti-mGr-1, APC anti-mLy-6C, or PerCP*-*Cy5.5 anti-mLy-6G (Biolegend). For some intracellular staining assays, cells were stained with FITC anti-mCD4, fixed and permeabilized with IC Fixation/Permeabilization buffer (eBioscience) for 20 min and then stained with PE anti-mIFN-γ, PE anti-mIL-4, PE anti-mIL-17A, PE anti-mFoxP3 or PE anti-mIL-10 (Biolegend). Cells were analyzed on FACS Canto II.

### Depletion of MDSCs with anti-Gr-1 antibody

Mice were intravenously injected with 150 μg/mouse anti-Gr-1 (RB6-8C5, Sanjian, Tianjin, China) at 0 and 3 day post CVB3 infection, and the depletion efficiency of cardiac infiltrating Gr-1^+^ cells at day 7 was >80% as determined by flow cytometry.

### Hormone treatment

Mice were administrated with 17β-Estradiol (Sigma) at a dose of 10 μg per mouse at day 1 post CVB3 infection as described previously^46^. Control mice received PBS with 0.5% ethanol without hormone.

### Isolation of MDSCs subsets

Hearts were aseptically removed from 8–10 CVB3-infected mice, perfused with 10 ml of warmed PBS and minced into 1 mm^3^ pieces. Following 4 h digestion with 0.25% trypsin, the debris was removed by strainer and supernatant was centrifuged and applied to Ficoll density gradient separation. Obtained infiltrated inflammatory cells were then stained with FITC anti-mCD11b, APC anti-mLy6C, and PerCP*-*Cy5.5 anti-mLy6G (Biolegend), and subjected to sorting using FACS Aria III. The purity of isolated CD11b^+^Ly6G^–^Ly6C^high^ monocytic MDSCs (M-MDSCs) and CD11b^+^Ly6G^+^Ly6C^low^ granulocytic MDSCs (G-MDSCs) was over 90% determined by FACS.

### T cell proliferation suppression assay

Myocardial M-MDSCs or G-MDSCs from CVB3-infected mice were seeded at 1 × 10^6^ per well. Sorted CD4^+^CD25^-^CD62L^high^CD44^low^ T cells (1 × 10^6^ per well) were added in the presence of purified anti-mCD3 (5 μg/ml, Biolegend) and anti-mCD28 (5 μg/ml, Biolegend) and culture for 72 h. And then cells were subjected to proliferation assays using cell proliferation ELISA BrdU kit (Roche) following the manufacturer’s instructions. Absorbance at 370 nm was detected on a microplate reader (Bio-Tek).

### Adoptive transfer of M-MDSCs

M-MDSCs were isolated from spleens of CVB3-infected female or male mice on day 7 post CVB3 infection and adoptively transferred via tail vein injection at a dose of 2 × 10^6^ cells per mouse into recipient male or female mice at day 3 post infection. Four days later, pathological change, inflammatory cell number as well as 7-day survival rates of recipient mice were examined.

### Myocardial viral load evaluation

Myocardial viral load on day 7 post infection was detected by real-time PCR assays as described previously^49^.

### Statistical analysis

Data are presented as mean ± SEM. Differences between experimental groups were analyzed for statistical significance by one-way ANOVA test followed by Tukey’s post hoc test or two-way ANOVA test. Survival rates of different groups were analyzed by log-rank test using GraphPad Prism version 4.01, (GraphPad Software Incorporated). A value of *p* < 0.05 was considered significant.

## Additional Information

**How to cite this article**: Su, N. *et al*. Monocytic myeloid-derived suppressor cells from females, but not males, alleviate CVB3-induced myocarditis by increasing regulatory and CD4^+^IL-10^+^ T cells. *Sci. Rep.* **6**, 22658; doi: 10.1038/srep22658 (2016).
